# Association of the HLA-DQA1 and HLA-DQB1 Alleles in Type 2 Diabetes Mellitus and Diabetic Nephropathy in the Han Ethnicity of China

**DOI:** 10.1155/2013/452537

**Published:** 2013-02-26

**Authors:** Ze-Jun Ma, Pei Sun, Gang Guo, Rui Zhang, Li-Ming Chen

**Affiliations:** 2011 Collaborative Innovation Center of Tianjin for Medical Epigenetics, Key Laboratory of Hormone and Development (Ministry of Health), Metabolic Disease Hospital & Tianjin Institute of Endocrinology, Tianjin Medical University, Tianjin 300070, China

## Abstract

HLA gene system is one of the most polymorphic regions of the human genome. The association of HLA class II genes in T1DM pathogenesis has been reported for several ethnicities. Associations of HLA class II genes with T2DM have revealed inconsistent results. Moreover, correlations between DN and HLA alleles remain unclear. We carried out DNA typing chip by specific medium resolution typing probes in 310 T2DM subjects (including 210 patients with DN and 100 patients without DN) in addition to 100 healthy controls. Differences were found between patients with T2DM and the control group in the frequencies of the HLA-DQA1∗0301 (15.5% versus 8.0%, *P* < 0.01) and the HLA-DQA1∗0501 alleles (16.6% versus 8.5%, *P* < 0.01). Differences were found between patients with DN and without DN in the frequencies of the HLA-DQA1∗0302 (6.9% versus 13.5%, *P* < 0.01) and HLA-DQB1∗0501 alleles (5.8% versus 14.5%, *P* < 0.01). Diabetes duration and systolic blood pressure were independent risk factors associated with DN (OR = 2.277 and 1.366, resp., *P* < 0.05), whereas the HLA-DQB1∗0501 llele had a protective effect on DN (OR = 0.53, *P* < 0.05). These data suggest the HLA-DQA1∗0301 and HLA-DQA1∗0501 alleles are markers of susceptibility for T2DM, and the HLA-DQB1∗0501 allele is associated with a protective effect on DN in Han ethnicity of China.

## 1. Introduction

Diabetic nephropathy (DN) is the leading cause of end-stage renal disease (ESRD) worldwide [1]. It affects approximately 30% of patients with long-standing Type 1 diabetes mellitus (T1DM) and Type 2 diabetes mellitus (T2DM) [2] and confers added risks of cardiovascular disease and mortality. Asians, including Chinese T2DM patients, have a higher prevalence of nephropathy, with 20% having clinical proteinuria and 40% microalbuminuria [3]. Multiple causes have been implicated in DN including hyperglycaemia, hypertension, inflammation, smoking, and dyslipidaemia [4, 5]. In addition, familial clustering of DN suggests genetic predisposition plays a role in the pathogenesis of this complication.

The HLA gene system is one of the most polymorphic regions of the human genome and one of the most extensively studied regions because of the association of variants at this locus with autoimmune, infectious, and inflammatory diseases [6]. It was proposed that both susceptible and protective alleles at the HLA DRB1, DQA1, and DQB1 loci were associated with the pathogenesis of some diseases [7]. T1DM is an autoimmune disease characterized by the selective destruction of pancreatic islets. Both genetic and environmental factors participate in the pathogenesis of the autoimmune process leading to the onset of this disease. T2DMis a complex heterogeneous group of metabolic disorders including hyperglycemia and impaired insulin action and/or insulin secretion, in which genetic factors play a complex and yet not clearly defined role. The association of HLA class II genes in T1DM pathogenesis has been reported for several ethnicities [8–10]. Studies on the association of HLA class II with T2DM have revealed inconsistent results, since an association [11], no association [12], and a weak link between HLA class II and T2DM have all been reported. Moreover, correlations between DN and HLA alleles remain unclear; the purpose of this study was to examine whether the HLA-DQA1 and HLA-DQB1 alleles are associated with the etiology of T2DM and DN in the Han ethnicity of China using the gene chip technique. 

## 2. Materials and Methods

### 2.1. Study Population

We selected T2DM subjects (*n* = 310) in Han ethnic group in the city of Tianjin, China, who were in-patients at the Department of Diabetic Nephropathy at Tianjin Medical University Affiliated Metabolic Disease Hospital. T2DM was diagnosed according to the 2007 American Diabetes Association diagnostic criteria. Exclusion criteria for this study were (1) diabetes secondary to known causes such as chronic pancreatitis and Cushing's syndrome and (2) T1DM defined by presentation with ketoacidosis or requirement of insulin therapy from the disease onset or positive GAD, IA-2A, or ICA antibody. Subjects were divided into groups with DN (DN1) and without DN (DN0) according to their 24 h albumin excretion rates (AERs). The group without DN (*n* = 100) consisted of patients who had been diagnosed with T2DM for at least 10 years and did not show albuminuria (AER < 30 mg/24 h). After ruling out urinary tract infection, hematuria, nephritis, and other conditions [11], the DN group was further subdivided into microalbuminuria group (*n* = 104, 300 mg/24 h > AER ≥ 30 mg/24 h) and overt albuminuria group (*n* = 106, AER ≥ 300 mg/24 h), with AERs determined in at least two consecutive overnight samples collected over a 3-to-6 month period. 

Controls consisted of healthy individuals including 50 males and 50 females (mean age 57.5 ± 4.7 yr). For inclusion in the study, control subjects had normal fasting/random glucose levels and no family history of DM or other autoimmune diseases. All were of Han ethnicity of China. 

### 2.2. Methods

Blood samples from all subjects were collected after obtaining written informed consent. The protocol was approved by the Ethics Committee of the Tianjin Medical University.

Peripheral blood samples were collected using EDTA-coated vacutainers. DNA was isolated according to the protocol of Sambrook et al. [13]. Unsymmetrical PCR was used to amplify the HLA-DQA1 and HLA-DQB1 exon 2 and exon 3. The amplification reactions contained 40 ng of DNA, 10 *μ*M dNTP mix, 1X PCR buffer (Applied Biosystems), 2 U of Taq polymerase (AmpliTaq Gold, Applied Biosystems), and 1 *μ*M of each primer in a total volume of 10 *μ*L. Each PCR was optimized with respect to the concentration of Mg^2+^ ions. Reactions were carried out in an ABI Gene Amp PCR system 9700. We carried out DNA-typing chip by specific medium resolution typing probes designed according to the gene frequency of HLA-DQA1 and HLA-DQB1 [14]. The PCR products were labeled and hybridized with the probes on the chip. The gene typing of HLA-DQA1 and HLA-DQB1 was certified by scanning the hybridized products and analyzing the read-out with Perkin Elmer Scanarray 4000 software.

### 2.3. Statistical Analysis

The clinical and laboratory characteristics were expressed as means ± SD. Observed distributions of genotypes were analyzed for deviation from the Hardy-Weinberg equilibrium by chi-square (*χ* ^2^) tests. Because there were no differences in the HLA genotype frequencies between the microalbuminuria and overt albuminuria groups, we combined these two groups into a DN group for further data analyses. Comparisons between the diabetes with and without DN groups and those between the genotypic groups were performed with unpaired Student's *t*-tests and *χ* ^2^ analysis. To evaluate the independent contributions of the HLA genotype to the risk of DN, we performed multivariate logistic regression analysis; the analyses included possible confounders (age at diagnosis of diabetes, diabetes duration, hypertension, triglyceride level, total cholesterol level, and A1C). Odds ratios (ORs) and 95% CIs were calculated. All calculations were performed using SPSS, Version 13.0 for Windows (SPSS, Chicago, IL, USA). We considered *P* < 0.05 as statistically significant in all analyses.

## 3. Results

Clinical and biochemical parameters of the controls and diabetic subjects are summarized in Table 1. The BMI, DBP, SBP, FBG, HbA1c, and triglyceride levels were found to be significantly high (*P* < 0.001) in diabetic patients than controls. The patients with DN differed significantly with respect to age at diagnosis of diabetes, diabetes duration, blood pressure, and triglyceride level compared to the group without DN (*P* < 0.05, *P* < 0.01, *P* < 0.01, and *P* < 0.01, resp.). 

Tables 2 and 3 show the frequencies of the HLA-DQA1 and HLA-DQB1 alleles between T2DM and control groups. Significant differences were detected between patients with T2DM and controls in the frequencies of the HLA-DQA1*0301 (15.5% versus 8.0%, *χ* ^2^ = 7.182,  *P* < 0.01) and HLA-DQA1*0501 alleles (16.6% versus 8.5%, *χ* ^2^ = 7.967, *P* < 0.01). The frequency of other HLA-DQB1 alleles in T2DM group did not differ from control group (*P* > 0.05). After controlling for confounding variables (including age, gender, BMI, FBG, HbA1c, and age at diagnosis of DM), logistic regression analysis demonstrated that HLA-DQA1*0301 and HLA-DQA1*0501 alleles remained significantly associated with T2DM (OR = 1.965, *P* < 0.01; OR = 2.137, *P* < 0.01, resp.) (Table 4).

Tables 5 and 6 show the frequencies of the HLA-DQA1 and HLA-DQB1 alleles between the DN and non-DN groups. There were decreased frequencies of the HLA-DQA1*0302 and HLA-DQB1*0501 alleles in DN group compared to the non-DN group (6.9% versus 13.5%, *χ* ^2^ = 7.172, *P* = 0.007; 5.8% versus 14.5%, *χ* ^2^ = 12.45, *P* = 0.004, resp.).

To evaluate the independent contributions of the polymorphism to the risk of DN, multivariate logistic regression analyses of T2DM patients with and without DN were performed by integrating the possible confounders in Table 7. After adjusting for factors such as aging, triglyceride levels, hypertension, and diabetes duration by using a logistic regression model, diabetes duration and systolic blood pressure were identified as independent and significant determinants of DN in these Chinese T2DM patients (OR = 2.277, *P* < 0.05; OR = 1.366, *P* < 0.05, resp.). The significance was retained for the HLA-DQB1*0501 allele (*P* < 0.05), but lost for the HLA-DQA1*0302 allele (*P* > 0.05).

## 4. Discussion

According to the China National Diabetes and Metabolic Disorders Study, the prevalence of diabetes in China in 2009 was 9.7% [15]. Over 90% of the Chinese diabetes patients are T2DM. Western lifestyle contributes a lot to the T2DM epidemic, and genetic determinants also influence T2DM susceptibility. Several genes involved in the affected metabolic pathways of T2DM have been regarded as candidates [16]. Additionally, recent data has indicated a role for certain HLA alleles in the pathogenesis of T2DM [11, 17]. To investigate the possible relationships between T2DM and the HLA alleles in the Han ethnic group, we analyzed these parameters in individuals of the same ethnicity in the city of Tianjin, China. The T2DM patients in this study showed a significant association with certain HLA-DQA1 alleles. The frequencies of the HLA-DQA1*0301 and HLA-DQA1*0501 alleles in T2DM group were 15.5% and 16.6%, respectively, which were significantly higher than those in normal group (approximately 8%); logistic regression analysis revealed that HLA-DQA1*0301 and HLA-DQA1*0501 alleles were nominally associated with susceptibility to T2DM.

The association of specific HLA genotypes with T2DM susceptibility/protection depends on the ethnicity and racial background of each population. Studies investigating the HLA-T2DM relationship are very limited, and the link between HLA and T2DM is still not conclusive. In two studies, using different genotyping methods, no association was found between T2DM and the HLA class II antigens (HLA-DR, HLA-DQ) in Punjabi Sikhs [19], while a positive association with HLA-DQA genes was reported for Belgians [20]. In Bahrainis, a population with a high prevalence of T2DM, T2DM was found significantly associated with both HLA-DRB1 and HLA-DQB1 genotypes, with some alleles appearing to confer susceptibility and others playing a protective role [11]. The inconsistencies reported in these studies may be accounted for by many potential factors, such as study design and sample size. Our finding that the HLA-DQA1*0301 and HLA-DQA1*0501 alleles are associated with the T2DM further complicates interpretation of these heterogeneous findings. Further studies in Chinese people with prospective data and more information on environmental factors are needed to better elucidate the effects of these genetic variants on diabetes risk and their interaction with environment. 

DN is a serious microvascular complication of diabetes, and it is a leading cause of end-stage renal disease in Western countries and in China. Traditionally, metabolic and hemodynamic factors are the main causes of renal lesions in patients with T2DM and DN, and although several genetic and environmental factors are likely to contribute to its development and progression, the precise mechanism for this contribution is still unknown. Specific ethnic populations are at unusual high risk for developing kidney disease. Native Americans (the Pima Indians of the southwestern United States) [21] and African Americans have a high prevalence of diabetes and kidney disease [22]. The reasons for the increased risk have not been clearly identified, but it is likely that there is a very strong genetic basis for the observed susceptibility, although factors such as hyperglycemia and hypertension also may play an important role. In any event, a concerted effort to understand the specific susceptibility of these groups is of great importance to the prevention of the increased morbidity and mortality associated with the onset of DN. 

When we examined the T2DM group who did not develop nephropathy, despite many years of diabetes, we found decreased frequencies of the HLA-DQA1*0302 and HLA-DQB1*0501 alleles compared to the non-DN group. Multivariate logistic regression analyses revealed that diabetes duration and systolic blood pressure were independent risk factors for the occurrence of chronic kidney disease in these patients and that the HLA-DQB1*0501 allele had a protective effect for DN. The existence of carriers of the HLA-DQB1*0501 allele with normoalbuminuria, despite long duration of diabetes, suggests that this allele is associated with reduced risk of DN. It is well known that chronic hyperglycaemia and the duration of diabetes are the most important risk factors for DN. DN progresses in some patients, despite good glycaemic control. Also, poor glycaemic control does not always lead to DN in younger onset patients, while still others develop severe DN. These facts suggest that the risk factors for DN are not necessarily the same among different patients and that the occurrence of DN may be influenced by genetic factors. Overall, our study indicates that the HLA-DQB1*0501 allele is associated with a protective effect against DN in the Han ethnicity of China. The mechanism underlying this protective association is still unknown, and further investigation of its functional role is needed.

## 5. Conclusion

The HLA-DQA1*0301 and HLA-DQA1*0501 alleles are markers of susceptibility for T2DM in the Han ethnicity of China, and the HLA-DQB1*0501 allele is associated with a protective effect on DN.

## Figures and Tables

**Table 1:** Clinical and biochemical parameters of the controls and diabetic subjects.

	T2DM patients		
	Without DN group	DN group	Controls
*N*	100	210	100
Age	57.8 ± 9.5	58.1 ± 8.7	57.5 ± 4.7
Gender (male/female)	47/53	105/105	50/50
Age at diagnosis of DM (years)	53.3 ± 10.5	51.7 ± 8.3^#^	—
Diabetes duration (years)	13.2 ± 2.7	16.1 ± 4.5^†^	—
BMI (kg/m^2^)	25.5 ± 3.6*	25.8 ± 4.0*	23.1 ± 3.8
Hypertension (%)	47 (47%)*	152 (72.4%)^†∗^	23 (23%)
SBP (mmHg)	136.2 ± 16.5*	144.1 ± 17.6^†∗^	130.4 ± 15.7
DBP (mmHg)	80.7 ± 9.5*	83.2 ± 9.6^†∗^	76.1 ± 8.9
FPG (mmol/L)	9.3 ± 5.8*	9.5 ± 6.4*	5.1 ± 3.7
HbA1c (%)	9.2 ± 4.3*	9.3 ± 5.7*	5.2 ± 2.8
Triglyceride level (mmol/L)	1.4 ± 0.7*	1.8 ± 0.9^†∗^	1.1 ± 0.6
Total cholesterol level (mmol/L)	4.8 ± 1.2	4.9 ± 1.4	4.7 ± 1.5

Data are expressed as means ± SD and *n* (%); *P < 0.01 versus controls; ^#^ *P *< 0.05 versus without DN group; ^†^ *P* < 0.01 versus without DN group.

**Table 2:** Frequency of HLA-DQA1 alleles in T2DM group versus control group.

HLA-DQA1 alleles	T2DM (*n* = 310)		Control (*n* = 100)			
	PN	AF	PN	AF	*χ* ^2^	*P*
DQA1*0101	50	0.081	18	0.090	0.174	0.677
DQA1*0102	65	0.105	20	0.100	0.038	0.845
DQA1*0103	62	0.100	19	0.095	0.042	0.837
DQA1*0104	50	0.081	20	0.100	0.726	0.394
DQA1*0201	43	0.069	21	0.105	2.670	0.102
DQA1*0301	96	0.155	16	0.080	7.182	0.007^(∗)^
DQA1*0302	56	0.090	24	0.120	1.513	0.219
DQA1*0401	46	0.074	22	0.110	2.549	0.110
DQA1*0501	103	0.166	17	0.085	7.967	0.005^(∗)^
DQA1*0601	49	0.079	23	0.115	2.442	0.118

PN: positive number; AF: antigen frequency; ^(∗)^ *P* value < 0.05.

**Table 3:** Frequency of HLA-DQB1 alleles in T2DM group versus control group.

HLA-DQB1 alleles	T2DM (*n* = 310)		Control (*n* = 100)			
	PN	AF	PN	AF	*χ* ^2^	*P*
DQB1*0201	58	0.094	15	0.075	0.642	0.423
DQB1*0301	64	0.103	16	0.08	0.927	0.336
DQB1*0302	114	0.184	36	0.18	0.015	0.902
DQB1*0303	132	0.213	50	0.25	1.205	0.272
DQB1*0401	53	0.086	16	0.08	0.059	0.808
DQB1*0501	54	0.087	19	0.095	0.116	0.733
DQB1*0601	89	0.144	32	0.160	0.325	0.568
DQB1*0602	56	0.09	16	0.08	0.201	0.654

PN: positive number; AF: antigen frequency.

**Table 4:** Logistic regression analysis of association between T2DM and HLA alleles.

Independent variables	OR	95% CI	*P *
HLA-DQA1*0301	1.965	1.235–3.135	0.009
HLA-DQA1*0501	2.137	1.461–2.816	0.007

OR: odds ratio; significant if *P* value < 0.05.

**Table 5:** Frequency of HLA-DQA1 alleles in T2DM patients with and without DN.

HLA-DQA1 alleles	DN0 (n = 100)		DN1 (n = 210)			
	PN	AF	PN	AF	χ^2^	P
DQA1*0101	16	0.080	34	0.081	0.002	0.968
DQA1*0102	21	0.105	44	0.105	2.862	0.091
DQA1*0103	19	0.095	43	0.102	0.082	0.775
DQA1*0104	11	0.055	39	0.093	2.619	0.106
DQA1*0201	12	0.060	31	0.074	0.400	0.527
DQA1*0301	27	0.135	69	0.164	0.888	0.346
DQA1*0302	27	0.135	29	0.069	7.172	0.007^(∗)^
DQA1*0401	10	0.050	36	0.086	2.516	0.113
DQA1*0501	38	0.190	65	0.155	1.214	0.270
DQA1*0601	19	0.095	30	0.071	1.034	0.309

PN: positive number; AF: antigen frequency; DN0: without DN group; DN1: DN group; ^(∗)^ *P* value < 0.05.

**Table 6:** Frequency of HLA-DQB1 alleles in T2DM patients with and without DN.

HLA-DQB1 allele	DN0 (*n* = 100)		DN1 (*n* = 210)			
	PN	AF	PN	AF	*χ* ^2^	*P*
DQB1*0201	18	0.09	40	0.095	0.044	0.834
DQB1*0301	20	0.10	44	0.105	0.033	0.855
DQB1*0302	32	0.16	82	0.195	1.121	0.290
DQB1*0303	41	0.205	91	0.217	0.110	0.740
DQB1*0401	16	0.08	37	0.088	0.114	0.736
DQB1*0501	29	0.145	25	0.058	12.45	0.004^(∗)^
DQB1*0601	24	0.12	65	0.155	1.332	0.249
DQB1*0602	20	0.1	36	0.086	0.337	0.562

PN: positive number; AF: antigen frequency; DN0: without DN group; DN1: DN group; ^(∗)^ *P* value < 0.05.

**Table 7:** Independent risk factors of DN in diabetic patients.

Independent variables	OR	95% CI	*P *
Diabetes duration	2.277	1.180–4.39	0.014
SBP	1.366	1.149–1.779	0.021
HLA-DQB1*0501	0.53	0.296–0.85	0.036

OR: odds ratio; significant if *P* value < 0.05.
